# The correlation between the presence of viremia and clinical severity in patients with enterovirus 71 infection: a multi-center cohort study

**DOI:** 10.1186/1471-2334-14-417

**Published:** 2014-07-29

**Authors:** Hao-Yuan Cheng, Yi-Chuan Huang, Ting-Yu Yen, Shao-Hsuan Hsia, Yu-Chia Hsieh, Chung-Chen Li, Luan-Yin Chang, Li-Min Huang

**Affiliations:** Department of Pediatrics, National Taiwan University Hospital and College of Medicine, National Taiwan University, Taipei, Taiwan; Department of Pediatrics, Kaohsiung Chang Gung Memorial Hospital and Chang Gung University College of Medicine, Kaohsiung, Taiwan; Department of Pediatrics, Children’s Hospital, China Medical University and Hospitals, Taichung, Taiwan; Department of Pediatrics, Chang Gung Memorial Hospital and Chang Gung University College of Medicine, Taoyuan, Taiwan

**Keywords:** Enterovirus, Viremia, Severe EV71 infection

## Abstract

**Background:**

Enterovirus 71 (EV71) is a great disease burden across the whole world, particularly in Southeast Asia. However, in recent decades, the pathogenesis of severe EV71 infection was not well understood. This study was aimed to investigate the correlation between the presence of viremia and the clinical severity of EV71 infection.

**Methods:**

We organized a prospective cohort study and enrolled laboratory-confirmed EV71 cases in six tertiary care hospitals in Taiwan during the EV71 epidemic from 2011 to 2012. Blood samples were collected once in the acute stage, on the first day of admission. We used real-time RT-PCR to detect EV71 viremia. Demographical and clinical data were collected and the clinical severity was categorized into four grades. Data analysis was performed to identify the risk factors of viremia and the correlation between viremia and clinical severity of EV71 infection.

**Results:**

Of the total 224 enrolled patients, 59 (26%) patients were confirmed to have viremia. Two-thirds (68%) of viremic cases were detected within the first three days of infection. Viremia occurred more frequently in children under the age of one year old (odds ratios [OR] 4.82, *p* < 0.001) but the association between the presence of viremia and complicated EV71 infection was not found (OR 1.02, p = 0.96). In the viremia group, patients had significantly more severe complications if viremia was detected after the third day of disease onset (26% vs. 5%, *p* = 0.03).

**Conclusions:**

Viremia occurred more frequently in children under the age of one year and viremia detected beyond three days after the onset of disease correlated with more severe disease in EV71 patients.

**Electronic supplementary material:**

The online version of this article (doi:10.1186/1471-2334-14-417) contains supplementary material, which is available to authorized users.

## Background

In recent decades, enterovirus infection has accounted for several outbreaks and has become a heavy burden of public health and socioeconomics in both developing and developed countries [1]. Among the numerous serotypes of enterovirus, enterovirus 71 (EV71) infection has especially drawn great attention because of the poor prognosis of severe infection [2, 3], possible neurologic sequelae [4–6] and high transmissibility [1, 7].

In Southeast Asia, outbreaks of EV71 infection have become a serious public health problem [1–3, 8, 9]. In Taiwan, EV71 has become one of the most significant epidemic diseases in children after the first documented large outbreak in 1998 [2, 4–6, 10, 11]. EV71 epidemics recur in Taiwan in intervals of three to four years and generate a great disease burden [12–16].

After investigating the characteristics of the transmission of EV71, the professionals in Taiwan’s Enterovirus Ad Hoc Committee and the Taiwan Centers for Disease Control designed several health policies, such as both physician and virological laboratory surveillance systems and hand-washing education program to control the epidemics of EV71. The above policies limited the outbreak and the impact of EV71 infection and reduced the numbers of fatal EV71 infection [15, 17]. As for the treatment of EV71 infection, a stage-based management decreased the mortality rate in EV71 patients as well [18]. However, regarding the pathogenesis of severe EV71 infection, research has not identified the mechanism causing severe EV71 infection and its complications [19]. Host factors, environmental factors and viral factors have been investigated for a long time but no conclusion has been achieved [13, 19–22]. This problem impedes the progress of the development of a more focused treatment strategy. Antiviral agents, such as pleconaril, have been studied in some in-vitro researches and clinical trials, but the efficacy of treating EV71 has not been confirmed [23, 24]. On the other hand, intravenous immunoglobulin was also used to treat severe EV71 infection based on its ability of virus neutralization and anti-inflammation, but the effectiveness was only based on retrospective researches [8, 18]. It is crucial for the development of EV71-specific treatment to test whether a new antiviral drug or an immunomodulator is effective. The study of EV71 viremia might provide more details about the pathogenesis of severe EV71 infection and contribute to further interventional studies. Although a previous study in animal model discussed the kinetics of viremia and clinical diseases [25], no similar study has been conducted on humans. As a result, we aimed to investigate whether viremia is correlated to the clinical severity of EV71 infection and seek to provide more information for further management of severe EV71 infection.

## Methods

### Patients

According to the laboratory-based surveillance system of Taiwan CDC, the baseline proportion of EV71 among all the enterovirus isolates from patients with enterovirus infections was about 2% but the proportion increased sharply between 2011 and 2012 and peaked in 2012 (45%) [26]. As a result, we considered the years 2011 and 2012 as epidemic years for EV71. We prospectively enrolled the EV71 cases hospitalized in six tertiary hospitals during the epidemic of EV71 from 2011 to 2012. The geographical distribution of patients included northern, central and southern Taiwan. All the patients were less than 18 years old and were suspected to have an EV71 infection according to their clinical manifestation such as hand, foot, and mouth disease (HFMD), herpangina or febrile illness without obvious focus during enterovirus epidemics. The study was approved by the institutional review board at National Taiwan University Hospital. The written informed consent was obtained from the parents of the studied children.

EV71 infection of clinically-suspected cases was confirmed on the basis of the following laboratory methods: positive virus isolation of EV71, and/or positive EV71 identification by pan-enterovirus real-time RT-PCR followed by molecular serotyping. We collected the basic demographic data including age and gender, the date of the onset of disease, the date of blood sampling for EV71 virologic test, and their clinical manifestations and outcomes. According to their clinical manifestation and outcome, the clinical severity of the patients with EV71 infection was classified according to the following four categories at the hospital discharge [15]:

Grade 1: Uncomplicated HFMD, herpangina or febrile illness,

Grade 2: Mild central nervous system (CNS) involvement including myolonic jerk alone or aseptic meningitis,

Grade 3: Severe CNS involvement, such as encephalitis, encephalomyelitis or polio-like syndrome, but without cardiopulmonary failure.

Grade 4: CNS involvement complicated by cardiopulmonary failure.

Blood samples for basic laboratory tests including complete blood count and virologic test were collected only once on the first day of admission. Real-time RT-PCR was used for detection of virus RNA in the blood sample to confirm the presence of viremia and its magnitude according to the protocol mentioned as below.

### Virus isolation and serotyping

Throat swabs were submitted for virus isolation. Samples were inoculated into human embryonic fibroblast (MRC-5), LLC-MK2, HEp-2, and RD cell cultures. If enteroviral cytopathic effect involved more than 50% of the cell monolayer, the cells were scraped and indirect fluorescent antibody staining with panenteroviral antibody (Chemicon International, Inc., Temecula, CA) was performed to identify the enterovirus. These isolates would be further serotyped to be EV71 by an immunofluorescent assay using EV71-specific mono-clonal antibodies against viruses (Chemicon International, Inc., Temecula, CA).

### Pan-enterovirus real-time RT-PCR

Viral RNA extraction from throat swabs and sera was performed using an isolation kit (RNA extraction kit, Qiagen, Hilden, Germany), and reverse transcription was performed with first strand cDNA synthesis kit for RT-PCR (Invitrogen, Carlsbad, CA, USA) according to the manufacturer’s directions. Real time RT-PCR for pan-enterovirus was performed with the primers of 5′-TCCTCCGGCCCCTGAATG-3′ and 5′-AATTGTCACCATAAGCAGCCA-3′, the probe of 6FAM-AACCGACTACTTTGGGTGTCCGTGTTTCXT-PH. The detection limitation (sensitivity) was ≤ 10 copies of in vitro transcribed RNA for pan-enterovirus real-time RT-PCR [27].

If the result of pan-enterovirus real-time RT-PCR was positive, molecular serotyping was subsequently performed. The cDNA was sent for semi-nested PCR with primers used in a previous report [27], the PCR product was purified, and auto-sequencing was performed with the forward primer on a 3730 ABI automatic sequencer with BigDye Terminator v3.1 Termination Cycle Sequencing Ready Reaction Kit (Applied Biosystems, Perkin-Elmer, Foster City, CA). The serotype was identified to EV71 by comparing the partial VP1 sequence to those recorded in the public gene database containing VP1 sequences for the strains of EV71.

### Statistical analysis

Descriptive analysis was performed for the demographic data and other characteristics of the EV71 patients. Viral load was presented as log_10_ copies/ml. In univariate analysis, Student’s *t* test and Mann–Whitney *U* test were used for continuous variables and chi-square test or Fisher’s exact test was used for categorical variables, when appropriate, to identify the factors associated with viremia and the risk factors of complicated EV71 infection. In multivariate analysis, multiple logistic regression analysis was used. A two-tailed p value of 0.05 was considered statistically significant. Data were maintained in Microsoft Excel, Mac, 2011 (Bellevue, WA) and analyzed by using Stata 12.0 (Stata Corp, College Station, TX).

## Results

### Basic characteristics

From 2011 to 2012, we totally enrolled 224 patients with their sera collected in this study. The median age was 2.71 years old (range 0 to 15 years), and male to female ratio was 1.49 (Table 1). Eighty-eight (39%) patients with laboratory-confirmed EV71 infection had an uncomplicated disease (Grade 1), such as HFMD, herpangina or simple febrile illness and 61 percent had at least one complication noticed, mostly mild CNS involvement (101 of 224 patients, 45%, grade 2). Most patients with mild CNS involvement had myoclonic jerk alone (100 of 101 patients, 99%) while only one patient had aseptic meningitis. Thirty-five of 224 patients (16%) had severe complication of EV71 infection (grade 3 and grade 4). Among total 224 patients, most patients were discharged smoothly without sequelae. There were only three patients who had neurologic sequelae, and there were only two deaths (Table 1).

**Table 1:** **Age, gender and clinical severity of patients with EV71 infection**

	Case number (n = 224)	Percentage
**Age (median with range, years)**	2.71 (0–15)	
≤ 3 y/o	102	46%
> 3 y/o	122	54%
**Gender**		
Male	140	63%
Female	84	38%
**Clinical Severity (Grade 1 to 4)**		
Uncomplicated disease^a^	88	39%
Myoclonic jerk or aseptic meningitis	101	45%
Severe CNS involvement^b^ without cardiopulmonary failure	22	10%
Severe CNS involvement^b^ with cardiopulmonary failure	13	6%
**Outcomes**		
Complete recovery	219	98%
With sequelae^c^	3	1%
Death	2	1%

^a^Uncomplicated cases includes uncomplicated HFMD, herpangina or febrile illness.

^b^Severe CNS involvement includes encephalitis, encephalomyelitis or polio-like syndrome.

^c^Among the three patients with sequelae, one patient had cervical mye1itis and thoracic spine arachnoiditis and had flaccid paralysis of right arm, another had rhombencephalitis complicated with left arm paralysis and ventilator-dependence after dishcharge, the other had paralysis of left arm and lower limbs and also needed non-invasive positive pressure ventilation after discharge.

### EV71 viremia

Sera of 224 EV71 cases were collected during acute stage and real-time RT-PCR was performed for enterovirus to detect EV71 viremia. The day of blood sampling for EV71 viremia ranged from the first day after the onset of disease to the seventh day, mostly on the third day (median four days).

**Figure 1:**
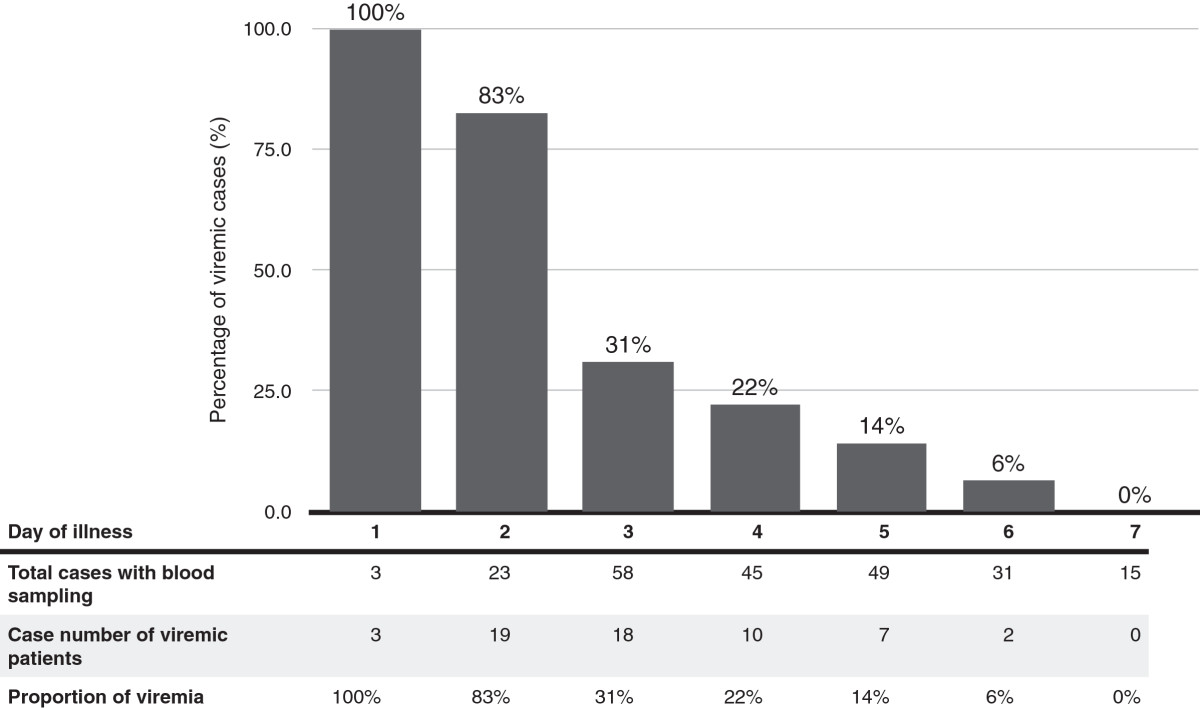
**The proportion of viremic patients with EV71 infection grouped by day of illness.**

**Figure 2:**
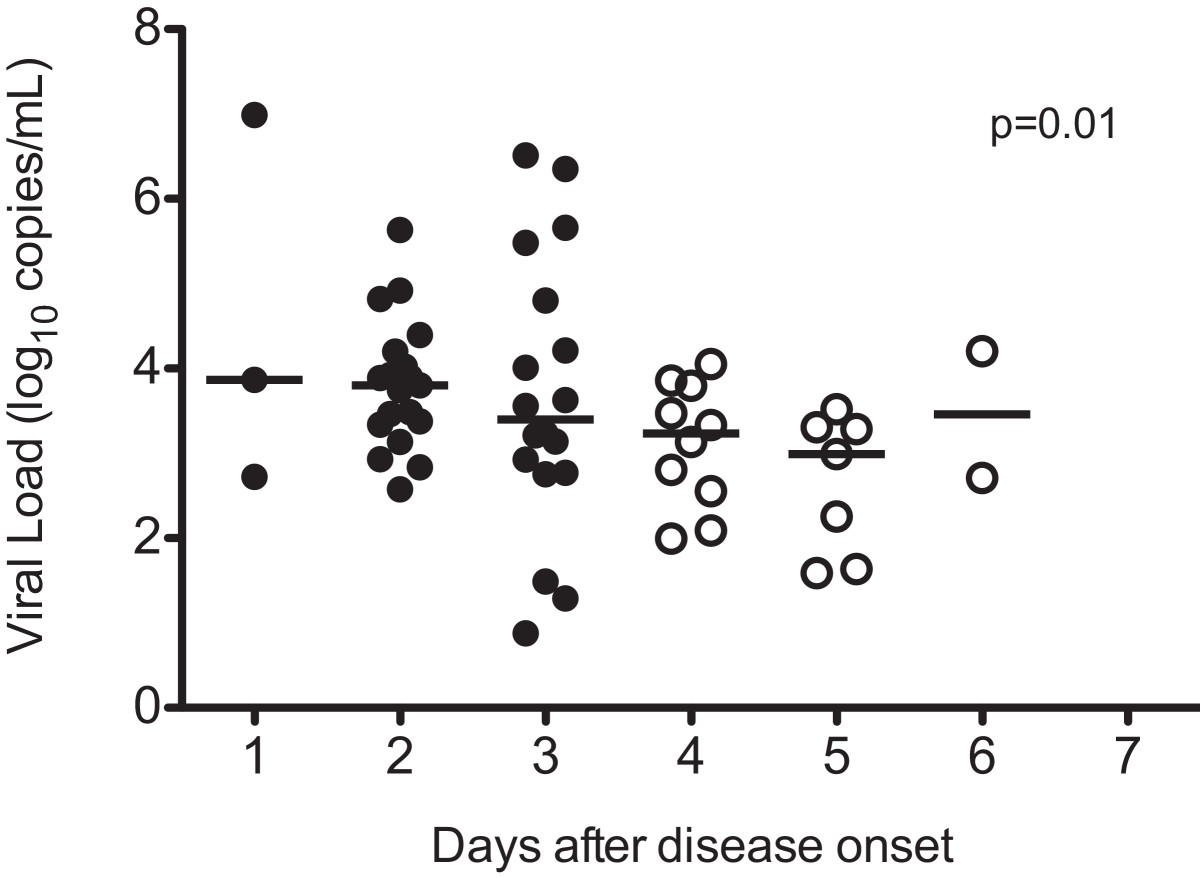
**Distribution of the levels of EV71 viremia in blood samples grouped by days after disease onset.** Day 1 indicates the first day of disease. The horizontal line presents the median viral load. Mann–Whitney *U* test was used for comparison between the serum viral loads sampled within and beyond three days after disease onset respectively.

Fifty-nine of 224 cases (26%) had positive PCR results and viremia was confirmed. The percentage of confirmed viremia was highest on the first day of illness and viremia was lower in patients sampled after day 3 (Figure 1). Two-thirds of viremic cases were detected within the first three days of disease (68% vs. 32%, respectively, *p* < 0.001). The viral load ranged from 0.88 log_10_ copies/mL to 6.99 log_10_ copies/mL (median 3.46 log_10_ copies/mL). The viral load was highest when the blood was sampled between the third and fourth day after disease onset, and the viral load was lower if the blood was sampled later (Figure 2). The viral load sampled during day 1 to day 3 was significantly higher than the viral load sampled on day 4 or later (median 3.68 vs. 3.13 log_10_ copies/mL, *p* = 0.01 with Mann–Whitney *U* test).

Children under one year of age were more likely to have viremia than children aged one or older (odds ratios [OR] 4.82, *p* < 0.001), while gender and other symptoms and signs, such as skin rash, oral ulcer, fever and the duration of fever, did not influence the presence or the magnitude of viremia (Table 2). Viremia did not have a significant effect on the distribution of clinical severity of EV71 cases. After we adjusted other factors in multivariate analysis, age under one year and the day of blood sampling were the only two significantly risk factors for EV71 viremia (Table 3).

**Table 2:** **Clinical characteristics of patients with and without EV71 viremia**

	No Viremia (n = 165)	Viremia (n = 59)	***p***value^a^
**Age (median with range, years)**	2.95 (0–14)	2.12 (0–15)	0.05
≤ 1 years old	10 (6%)	14 (24%)	<0.001
> 1 years old	155 (94%)	45 (76%)	
**Gender**			0.43
Male	106 (64%)	34 (42%)	
Female	59 (36%)	25 (58%)	
**Skin rash**	155 (94%)	58 (98%)	0.30
**Oral ulcer**	162 (98%)	55 (93%)	0.08
**Fever** ^**b**^	152 (92%)	52 (88%)	0.43
**Duration of fever (mean ± SD, days)**	2.99 ± 1.85	2.38 ± 1.99	0.07
**Clinical Severity (Grade 1 to 4)**			
Uncomplicated disease	65 (39%)	23 (39%)	0.54^d^
Complicated disease	100 (61%)	36 (61%)	
Myoclonic jerk or aseptic meningitis	72 (44%)	29 (49%)	
Severe CNS involvement without cardiopulmonary failure	17 (10%)	5 (8%)	
Severe CNS involvement with cardiopulmonary failure	11 (7%)	2 (3%)	
**Sample day for PCR test** ^**c**^			
≤ 3 days	44 (27%)	40 (68%)	<0.001
> 3 days	121 (73%)	19 (32%)	
**Viral load** ^**c**^ **(median with range, log**_**10**_ **copies/mL)**			
≤ 3 days		3.68 (0.88 - 6.99)	0.01
> 3 days		3.13 (1.56 – 4.20)	

Values expressed as n(%), unless otherwise indicated.

^a^The chi-square test was used for categorical variables and the Mann–Whitney test was used for comparison of the viral load.

^b^Fever was defined as any body temperature elevation over 38 degrees Celsius.

^c^Caterorized as days after disease onset.

^d^Statistical analysis was performed for the difference between patients with uncomplicated and complicated diseases.

**Table 3:** **Multivariate analysis for risk factors of EV71 viremia**

	Odds ratio	95% CI	***p***value
**Age <1 year**	4.27	1.45 - 12.59	0.008
**Sex (Male)**	0.98	0.82 - 1.18	0.84
**Skin rash**	0.70	0.21 - 2.40	0.57
**Fever** ^**a**^	7.81	0.40 - 152.76	0.18
**Oral ulcer**	0.34	0.02- 5.48	0.45
**Grade 2 to 4 EV71 disease**	1.16	0.54 - 2.47	0.70
**Day of illness on sampling (day)**	0.39^b^	0.28 - 0.54	<0.001

^a^Fever was defined as any body temperature over 38 degrees Celsius.

^b^The value represents the change in odds ratio per day after disease onset.

### Factors associated with clinical severity

In univariate analysis, no correlation between the presence of viremia and the clinical severity of EV71 infection was found (Table 4). No significant difference of the percentage of viremic cases was found between patients with mild diseases (grade 1 to 2) and with severe complications (grade 3 to 4) (27.5% and 20%, respectively, *p* = 0.41). The risk factor for complicated EV71 diseases was fever (OR 7.33, *p* < 0.001) and fever over 3 days (OR 2.49, *p = 0.002*). Higher viral load did not correlate with more severe clinical disease. Those patients with complicated EV71 infection were younger than those with uncomplicated disease (median age 2.60 vs. 3.08 years, *p* = 0.06). The multivariate analysis adjusting age, gender, and clinical symptoms revealed similar results (Table 5).

**Table 4:** **Predictive factors of complicated diseases in patients with EV71 infection**

	Uncomplicated EV71 infection (Grade 1) (n = 88)	Complicated EV71 infection (Grade 2 to 4) (n = 136)	***p***value^a^
**Age (median with range, years)**	3.08 (0–15)	2.60 (0–14)	0.06
**Gender**			
Male	56 (64%)	84 (62%)	0.89
Female	32 (36%)	52 (38%)	
**Fever** ^**b**^	72 (82%)	132 (97%)	<0.001
**Fever > 3 days**	34 (39%)	83 (61%)	0.002
**Skin rash**	86 (97%)	124 (94%)	0.21
**Oral ulcer**	85 (97%)	132 (97%)	1.00
**Viremia**			
Yes	23 (26%)	36 (26%)	0.96
No	65 (74%)	100 (74%)	
**Viral load** (median with range, log10 copies/mL)	3.34 (0.88 - 6.99)	3.50 (1.29 - 6.51)	0.80

Values expressed as n(%), unless otherwise indicated.

^a^The chi-square and Fisher’s exact test was used for categorical variables, and the Mann–Whitney test was used for comparison of the viral load.

^b^Fever was defined as any body temperature over 38 degrees Celsius.

**Table 5:** **Multivariate analysis of risk factors of complicated EV71 disease (grade 2 to 4)**

	Odds ratio	95% CIs	***p***value
**Age <1 year**	2.57	0.87 - 7.62	0.09
**Sex (Male)**	0.99	0.86 - 1.15s	0.92
**Skin rash**	0.29	0.05 - 1.54	0.15
**Fever** ^**a**^	5.63	1.74 - 18.28	0.001
**Oral ulcer**	1.91	0.31 - 11.74	0.48
**Day of illness on sampling (day)**	1.05^b^	0.84 - 1.31	0.66
**Viremia**	1.26	0.61 - 2.59	0.54

^a^Fever was defined as any body temperature over 38 degrees Celsius.

^b^The value represents the change in odds ratio per day after disease onset.

**Figure 3:**
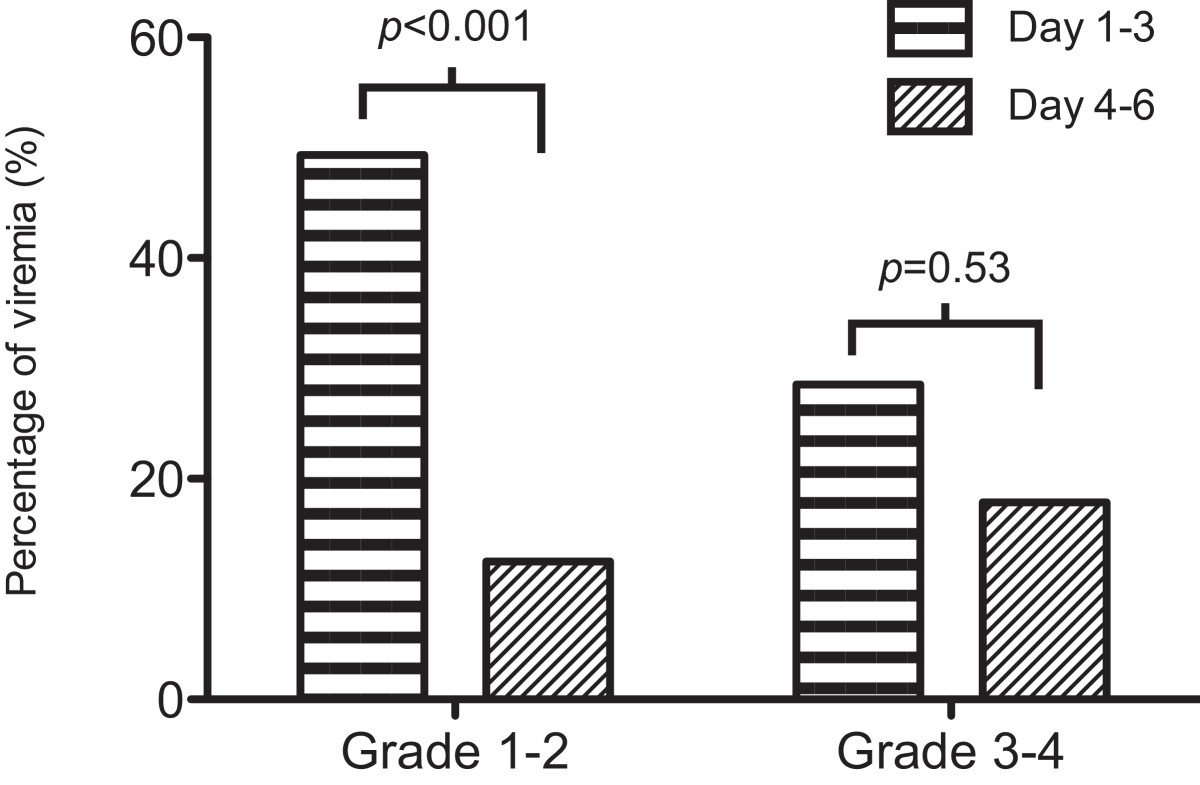
**The percentage of viremia within and beyond three days after disease onset in patients who had mild diseases (grade 1 to 2) and severe complications (grade 3 to 4).**

For those who had severe complications (grade 3 to 4), the percentage of viremic cases did not decrease beyond three days of illness compared to those who had milder EV71 diseases (grade 1 to 2) (Figure 3). Contrarily, in the viremia group, patients had significantly more severe complications if their viremia was detected after the third day of disease onset (26% vs. 5%, *p* = 0.03).

## Discussion

In our study, 224 patients with laboratory-confirmed EV71 infection were enrolled. Approximately one-fourth of the EV71 patients had viremia detected in acute stage of disease. To our knowledge, this is the first study to investigate the correlation between EV71 viremia and the clinical severity of EV71 infection and we found that prolonged viremia was associated with the risk of severe complicated course of EV71 infection.

As for EV71 infection, the classical clinical manifestation, disease course and possible impacts on neurologic and cognitive function has been documented thoroughly in previous studies [4, 21, 22, 28, 29]. In our study, patients with complicated EV71 infection accounted for about 60% of total EV71 cases. Most of them had only myoclonic jerk. Compared to previous epidemiology data [4, 7, 30, 31], the proportion of cases with severe CNS involvement was lower in our cohort. This reduction may be related to different cohorts or different hospital setting.

According to previous retrospective studies, the age of patients is an important risk factor of severe EV71 infection [16, 32]. Our study demonstrated similar tendency, that is, younger children were more vulnerable to have CNS involvement. We also found that children with age under one had a significantly higher rate of viremia, which might be correlated to more complicated clinical manifestations.

Most of the cases with viremia in this study were detected before the third day of disease. This tendency is compatible to the clinical observation about the mean duration of a prodromal stage with fever before the following neurologic complications caused by EV71 [4]. After the third day of disease onset, the proportion of patients with viremia decreased to around one-fourth although the proportion of complicated disease remained at the similar level. No viremia was detected after the seventh day of disease onset even if these patients had persistent clinical symptoms, indicating that the resolution of enterovirus viremia occurred most within seven days and that deterioration of the clinical disease beyond seven days was rare. In animal models, the viral kinetics in the systemic EV71 infection in rhesus monkeys also revealed a similar pattern compared to our study [25].

Additionally, the percentages of patients with CNS involvement in patients with or without viremia were similar and we did not find the positive correlation between the presence of viremia and the clinical severity of EV71 infection. Viremia is an interesting topic of virus infection and many researchers have taken great efforts to establish correlation between the clinical manifestation and the duration or the magnitude of viremia in several viral diseases. In some viral infections, such as the Epstein–Bar virus and cytomegalovirus disease [33–35], the presence or the magnitude of viremia may be able to predict clinical severity, while in other cases, such as dengue virus, norovirus and hantavirus, the presence of viremia usually only indicated the active stage of disease, but did not absolutely predict the clinical severity [36–39]. The observation in our study suggested that EV71 might be more similar to the later ones.

A possible explanation is that EV71 is considered to be a neurotropic infectious microorganism [40] and the presence of viremia is not the most crucial step for its severe infection. Viremia may only occur in a certain stage of EV71 infection for a short period and most resolve spontaneously soon afterwards. Another supporting evidence is that the virus can be detected from throat swab and rectal swab [19, 32, 41], which are the primary sites of EV71 infection and it is the cellular tropism that contributes to the clinical presentations and the following complications, for example, myoclonic jerk and other CNS involvement in EV71 infection.

Furthermore, we found that the proportion of detected viremic cases in patients with severe complications (grade 3 to 4) remained similar after the third day of disease while the proportion of viremia decreased in those who had mild diseases (grade 1 to 2). The finding suggested the viremia might persist longer in those who had severe diseases and thus their risk of systemic involvement and complications increased. In addition, the proportion of patients with severe CNS involvement and cardiopulmonary failure increased in those patients whose viremia was detected after the third day of disease. That is to say, a much higher virus burden, prolonged viremic phase or the consequent immunologic reactions might be the principle mechanism of the severe complications of disease rather than viremia alone. Previous studies suggested that the most severe form of EV71 infection causing pulmonary edema might result from the overreaction of immune system and consequent cytokine storm [19, 42]. Besides, a prospective cohort study performed in Sarawak during three EV71 outbreaks over seven years and our previous study [2, 8], showing that a fever for three days or longer was associated with neurological complications, may also support this hypothesis. On the other hand, a previous pathogenesis study performed in rhesus monkey has suggested CNS invasion was correlated with severe CNS diseases with pulmonary edema and the viral loads in the CNS were parallel to the viremia. As a result, a prolonged viremia may increase the viral load in the CNS and is more likely to lead to more severe CNS disease and further cardiopulmonary failure [25].

Nevertheless, the clinical severity of EV71 infection may be multifactorial and is not determined only by the presence of viremia. Either the different virulence of different genotypes, host factors and other socioeconomic factors [13, 19–22] may contribute to the severe complication and sequelae of EV71 infection as well.

There are some limitations in our study. First, we only collected blood samples for PCR test at one time point in each patient and it is difficult to describe the kinetics and the duration of viremia. A kinetic data could provide more information about the influence of viremia on the clinical manifestation, disease severity and the whole course of EV71 infection. It is also impossible to determine the peak viral load of individuals and this limitation makes it more difficult to clarify the definite relationship between the magnitude of viremia and the clinical manifestation. Second, most patients in our cohort had only uncomplicated disease or myoclonic jerk and the proportion of severe CNS complications or further cardiopulmonary failure was relatively low. Because the epidemics of non-polio enterovirus occurred almost every year in Taiwan, the decreased severity of EV71 infection may result from partial cross-protection from previous enterovirus infection by different serotypes or genotypes [43]. Only 15.6% of patients with EV71 infection in this study had severe complications. The sample size was not large enough to establish the correlation between viremia and severe complications of EV71 infection.

## Conclusions

In summary, our study found that viremia occurred more frequently in children under the age of one year and viremia detected beyond three days after the onset of disease correlated with more severe disease in EV71 patients. Our findings may shed a light on the identification of the role of viremia for the pathogenesis of severe EV71 infection, but further studies targeted at the interaction between the magnitude, the kinetics of viremia and other host factors in patients with severe EV71 infection will be required.

## Authors’ original submitted files for images

Below are the links to the authors’ original submitted files for images. Authors’ original file for figure 1 Authors’ original file for figure 2 Authors’ original file for figure 3
