# Supplementary figures and images for: The correlation between the presence of viremia and clinical severity in patients with enterovirus 71 infection: a multi-center cohort study
Source: BMC Infect Dis. 2014 Jul 29;14:417. doi: 10.1186/1471-2334-14-417 (PMC4133623; doi:10.1186/1471-2334-14-417)

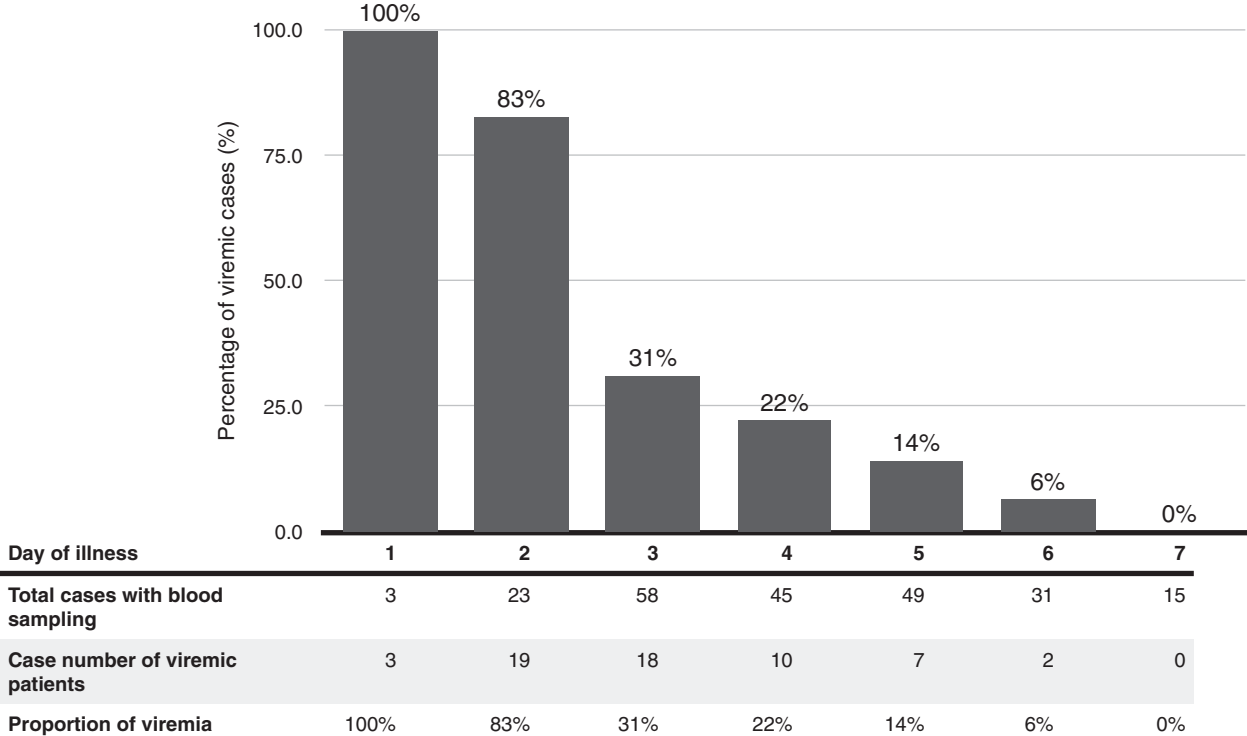

Supplement: Supplementary file 1 — Authors’ original file for figure 1 [file 12879_2014_3735_MOESM1_ESM.pdf]

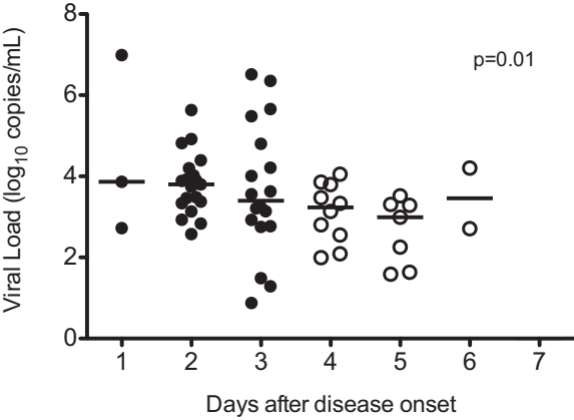

Supplement: Supplementary file 2 — Authors’ original file for figure 2 [file 12879_2014_3735_MOESM2_ESM.pdf]

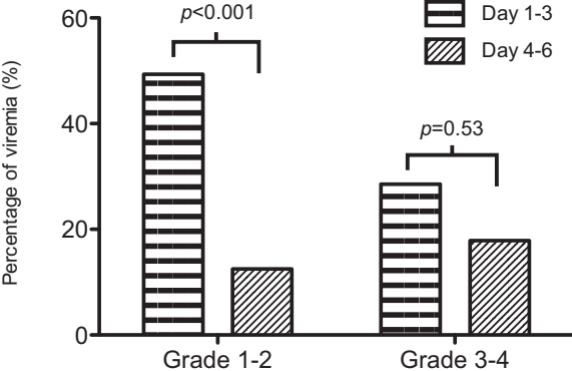

Supplement: Supplementary file 3 — Authors’ original file for figure 3 [file 12879_2014_3735_MOESM3_ESM.pdf]
